# Proton Radiotherapy for Patients With Oligometastatic Breast Cancer Involving the Sternum

**DOI:** 10.14338/IJPT-21-00014

**Published:** 2021-11-11

**Authors:** Andrew Johnson, Nicolas Depauw, Stephen Zieminski, Rachel Jimenez

**Affiliations:** Massachusetts General Hospital, Department of Radiation Oncology, Boston, MA, USA

**Keywords:** oligometastatic breast cancer, proton radiotherapy, pencil beam scanning

## Abstract

**Introduction:**

A subset of metastatic breast cancer patients present with oligometastatic disease involving the sternum. Given the proximity to traditional target structures, a proton radiation field can be expanded to include this region, providing definitive therapy for patients who are otherwise metastatic. We evaluated the feasibility and outcomes of a small series of patients who received comprehensive nodal irradiation inclusive of an isolated sternal metastasis using proton pencil beam scanning.

**Materials and Methods:**

Four patients with a diagnosis of metastatic breast cancer with an isolated metastasis to the sternum received multimodality therapy with curative intent and then underwent adjuvant pencil beam scanning with definitive treatment to the sternum. Dosimetric parameters and treatment outcomes were evaluated.

**Results:**

With respect to treatment coverage, proton therapy was able to deliver comprehensive target structure coverage while maintaining modest doses to the organs at risk compared with photon techniques. At a median follow-up of 28 months from diagnosis, none of the patients have experienced relapse within the radiation portal or developed additional sites of metastatic disease.

**Conclusion:**

Pencil beam scanning for oligometastatic breast cancer with isolated sternal lesions appears feasible without undue normal tissue exposure. Current treatment outcomes appear promising.

## Introduction

An estimated 1% to 10 % of patients who present with metastatic breast cancer have oligometastatic disease involving one or a few metastatic lesions [1]. As systemic therapy options improve for these patients, there is often a desire among multidisciplinary care teams to treat patients with oligometastatic breast cancer aggressively [2, 3]. A subset of oligometastatic patients with breast cancer present with disease limited to the sternum and radiation oncologists may be approached to treat with definitive intent using radiation treatment fields that cover not only the breast and regional lymphatics, but also the sternal metastasis. However, adequate radiation delivery to this area is often limited by normal tissue exposure to the heart, bilateral lungs, and contralateral breast.

Pencil beam scanning (PBS) is increasingly used for comprehensive breast radiotherapy, including treatment of the regional lymph nodes. Using intensity modulation, PBS improves treatment for patients by delivering complete target coverage of both the chest wall and/or breast along with the involved nodal regions, in particular the internal mammary lymph nodes, while substantially reducing dose to cardiac/lung structures [4]. PBS has the potential to be used for treatment of patients with oligometastatic disease involving the sternum without the same concerns for normal tissue exposure.

In this case series, we evaluated the following: (1) the feasibility of using PBS to treat traditional radiation fields inclusive of the site of oligometastatic disease in the sternum in patients with left-sided breast cancer, both in regard to target coverage and normal tissue exposure, and (2) the early clinical outcomes of these patients, including both side effects of radiation and freedom from disease progression.

## Case Report

**Figure 1.**
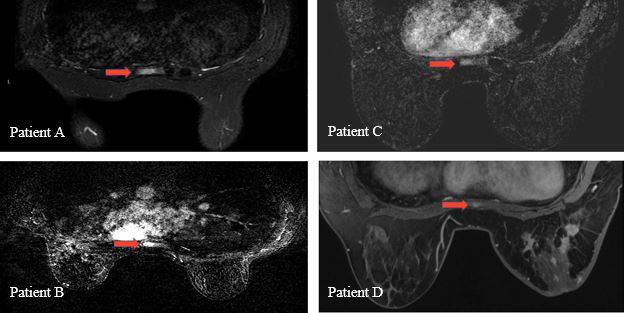
Diagnostic magnetic resonance imaging of oligometastatic sternal lesions (axial view) Red arrow indicates the location of the sternal lesion.

This case report examines 4 women (Patients A–D), median age 33 years (range, 29–44), diagnosed with de novo oligometastatic breast cancer to the sternum, all in 2018. Institutional review board approval was obtained for this study. Treatment characteristics are described in **Table 1**. All women underwent staging chest, abdomen, and pelvis as well as bone scan and breast magnetic resonance imaging (MRI) to give radiologic confirmation of metastatic disease originating in the left breast and limited to the sternum (**Figure 1**). All women then received treatment with definitive intent beginning with neoadjuvant chemotherapy. Patients A and C were HER2-neu negative and received anthracycline-based therapy while Patients B and D were HER2-neu positive and received Herceptin–Perjeta regimens. Patient A underwent lumpectomy as definitive surgical intervention and the remaining patients underwent mastectomy. All received axillary lymph node dissection. All patients demonstrated downstaging after neoadjuvant therapy with 3 of 4 demonstrating a complete response in the lymph nodes.

**Table 1.** Treatment characteristics for patients with oligometastatic sternal lesions from breast cancer receiving proton therapy.

Patient	**Age at diagnosis**	**Hormone receptor status**	**Clinical stage**	**Neoadjuvant chemotherapy regimen**	**Surgery**	**Adjuvant Chemotherapy regimen**	**Endocrine therapy**	**Radiotherapy**		
								Total dose	Dose per fraction	EQD2
A	36	ER−/PR−/HER2−	T3N1	AC-T	Lumpectomy	Capecitabine	None	60	2	60
B	44	ER+/PR−/HER2+	T4N1^a^	TCHP, HP	Mastectomy	Ado-trastuzumab emtansine	Letrozole + Lupron	60	2.4	65
C	29	ER+/PR+/HER2−	T3N1	AC-T	Mastectomy	Palbociclib	Letrozole + Lupron	60	2.4	65
D	30	ER+/PR+/HER2+	T3N1^b^	THP, AC	Mastectomy	Ado-trastuzumab emtansine	Tamoxifen + Lupron	60	2.4	65

Abbreviations: EQD2, equieffective dose; AC-T, Adriamycin–cyclophosphamide–Taxol; TCHP, Taxotere–carboplatin–Herceptin–Perjeta; HP, Herceptin–Perjeta; THP, Taxol–Herceptin–Perjeta.

a Patient B had sternal lesion suspicious for metastatic disease on magnetic resonance imaging (MRI) and consistent with metastasis on bone scan, but not considered amenable to performing a biopsy.

b Patient D had sternal lesion suspicious for metastatic disease on MRI and bone scan, fine-needle aspiration showed atypical cells that could not be further characterized.

**Figure 2.**
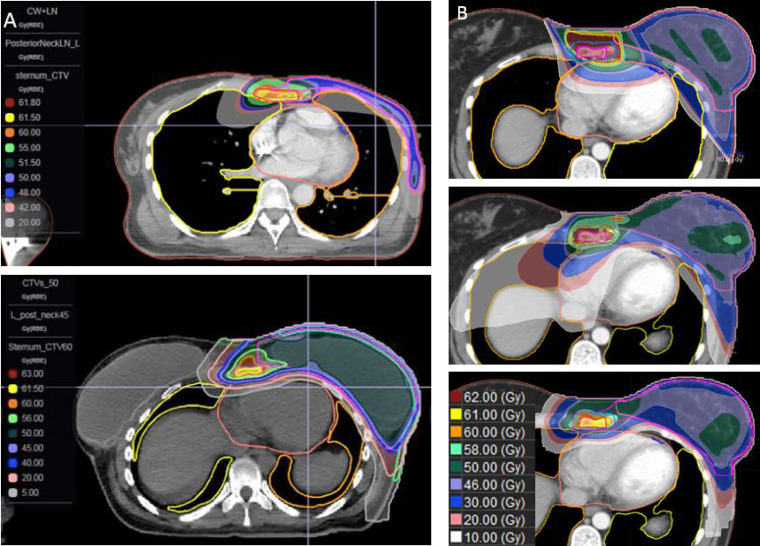
(A) Example pencil beam scanning (PBS) plans. (B) Example comparison plan of 3-dimesional (3D), volumetric modulated arc therapy (VMAT), and PBS. ([A] top) Depicts a postmastectomy plan without reconstruction (Patient B). ([A] bottom) Depicts a postmastectomy. plan with implant-based reconstruction (Patient C). ([B] top) 3D; ([B] middle) VMAT; and ([B] bottom) PBS (Patient A).

For radiation planning, all patients underwent a computed tomography–based simulation using 2.5-mm slices. The computed tomography scan was fused with each patient's preoperative MRI for localization of the oligometastatic site. The radiation oncologist contoured all clinical target volumes including the sternal lesion, chest wall or breast, and regional lymph nodes (axilla, supraclavicular region) as well as all organs at risk, including the heart, left anterior descending artery, and bilateral lungs. Proton plans were generated with the Astroid planning system (.decimal, LLC), using a multicriteria optimization Pareto-surface navigation to arrive at the most clinically suitable solution. The approach consisted of a single en face field on a proton treatment machine with energies ranging from 230 to 90 MeV and corresponding spot sizes of 8 to 14 mm. An 80-mm range shifter was used to ensure potential delivery to the patient's surface. Treatment plans were designed to simultaneously achieve adequate coverage of all targets while maximally sparing dose to all organs at risk. All patients were prescribed 60 Gy(RBE) to the site of sternal disease, with 3 of 4 receiving 60 Gy(RBE) in 2.4 Gy(RBE) fractions for an equieffective dose (EQD2) of 65 (**Table 1**). Prescription doses for all other targets were 45.0 to 50.4 Gy(RBE) or Gy(RBE) in daily 1.8 Gy(RBE) or 2 Gy(RBE) fractions for all plans. Representative PBS plans are depicted in **Figure 2A**. Across all patients, the sternum clinical target volumes received a mean and maximum dose of 60.1 and 62.2 Gy(RBE), respectively, and all other target structures received prescription doses, while still achieving modest mean doses to the heart of 1.83 Gy(RBE) and mean maximum doses to the left anterior descending artery of 7.5 Gy(RBE), across all 4 patients. For the bilateral lungs, mean doses were similarly low (**Table 2**). Comparison plans using both three-dimensional and volumetric modulated arc therapy techniques for patients A and D are provided for dosimetric comparison (**Table 3**, **Figure 2B**).

**Table 2.** PBS dosimetry information.

Patient	CTV_sternum		CTV_IMNs		CTV_chest wall		Heart		LAD		Left lung	Right lung
	Mean Gy	Max Gy	Mean Gy	Max Gy	mean Gy	Max Gy	Mean Gy	Max Gy	Mean Gy	Max Gy	Mean Gy	Mean Gy
A	58.86	61.70	46.17	51.70	45.27^a^	57.80	1.83	39.60	1.14	4.70	7.10	0.97
B	60.45	61.90	51.10	59.70	49.94	56.80	1.47	31.50	0.89	10.60	9.43	3.42
C	60.00	62.10	49.94	60.30	49.94	56.00	1.98	43.00	0.21	2.60	5.84	0.44
D	60.93	63.10	49.82	59.93	49.97	58.04	2.02	54.32	1.48	12.05	7.73	0.53

Abbreviations: CTV, clinical target volume; IMN, internal mammary lymph node; LAD, left anterior descending artery.

a Patient A underwent breast conserving surgery and received whole breast radiation prescribed to 45 Gy(RBE).

**Table 3.** Comparison dosimetry with photon techniques.

Technique	CTV_sternum		CTV_IMNs		CTV_breast/CW		Heart		LAD		Left lung	Right lung
	Mean Gy	Max Gy	Mean Gy	Max Gy	Mean Gy	Max Gy	Mean Gy	Max Gy	Mean Gy	Max Gy	Mean Gy	Mean Gy
Patient A												
3D	63.69	68.35	45.75	58.67	47.26	63.68	10.24	64.19	24.57	44.28	17.45	1.18
VMAT	62.21	66.01	48.32	53.53	50.53	63.85	9.77	59.88	8.28	15.38	11.24	9.24
PBS	58.86	61.70	46.17	51.70	45.27	57.80	1.83	39.60	1.14	4.70	7.10	0.97
Patient D												
3D	63.45	71.14	52.43	67.09	52.48	69.44	20.90	65.09	36.43	54.17	21.65	1.03
VMAT	61.69	64.97	52.52	62.20	52.22	63.67	15.44	61.99	12.03	23.06	13.98	10.13
PBS	60.93	63.10	49.82	59.93	49.97	58.04	2.02	54.32	1.48	12.05	7.73	0.53

Abbreviations: CTV, clinical target volume; IMN, internal mammary lymph node; CW, chest wall; LAD, left anterior descending artery; 3D, three dimensional; VMAT, volumetric modulated arc therapy; PBS, pencil beam scanning.

By the completion of radiation, all patients had developed grade 2 radiation dermatitis in the treatment field, more pronounced at the skin overlying the sternal site for 2 of 4 patients. Patient A developed grade 2 radiation esophagitis. None of the patients developed moist desquamation or any grade 3 toxicity. After PBS, all patients completed adjuvant therapy. Patients B through D had ER+ and/or PR+ positive cancers and were placed on endocrine therapy and ovarian suppression. Patients B and D completed a year of HER2-directed therapy, while patient A was triple negative and completed 6 months of Capecitabine. At a median follow-up of 28 months from diagnosis, all 4 patients remain without evidence of disease progression, both clinically and on surveillance imaging.

## Discussion

PBS for patients with oligometastatic left-sided breast cancer involving the sternum appears to be both feasible and effective despite varying tumor biology. This treatment approach may be particularly valuable for young patients, as presented in the case series above. As systemic therapy in the metastatic setting continues to improve for breast cancer, local control also becomes increasingly important, and this includes control of limited oligometastatic sites like the sternum that are not readily amenable to surgical resection.

Recent prospective studies have suggested a lack of survival benefit with the use of aggressive local therapy among patients with de novo stage IV breast cancer [5]; however, patients presenting with fewer sites of metastatic disease and those with bone only disease have also been shown to have superior survival [6, 7]. A single-institution retrospective series compared outcomes among 35 de novo metastatic breast cancer patients with disease limited to the mediastinum/sternum and treated with curative intent with stage IIIC breast cancer patients, a palliative cohort with disease isolated to the mediastinum/sternum and not treated with curative intent, and another palliative cohort of de novo metastatic disease. They found similar rates of 5-year local regional recurrence-free survival, recurrence-free survival, and overall survival between the mediastinum/sternum curative intent group and the stage IIIC cohort while both cohorts treated with palliative intent fared worse, suggesting that aggressive treatment with curative intent for those patients with disease limited to the sternum/mediastinum has value [8]. However, the majority of the 35 patients who received definitive therapy for de novo metastatic disease limited to the mediastinum/sternum received tangential field photon radiation and little information regarding dosimetry was provided. The authors do relay that those receiving an intensity-modulated radiotherapy or proton boost experienced a nonsignificant, but improved local control rate suggesting that comprehensive coverage of the tumor sites may be enhanced with advanced techniques that permit comprehensive treatment over what would be achievable using tangential fields alone. Our dosimetric comparisons highlight these tradeoffs, demonstrating that comprehensive coverage of the metastatic site and regional lymphatics with proton therapy need not come at the expense of excessively high doses to the heart and lungs, as would be required with photon techniques.

While this case series is too small to make definitive conclusions, it lends support to the report of Christopherson and colleagues [8] in that there may be something particularly unique biologically among patients who manifest with isolated sternal metastases, given its proximity to the primary tumor site, and this could provide a superior chance of successful salvage therapy. The SABR-COMET trial showed an improvement in overall and progression free survival among various cancer types including breast cancer when bone metastases were treated with conventional palliative dosing (EQD2 < 36, assuming alpha/beta ratio of breast cancer of 3) [9]. The currently accruing NRG BR002 is exploring the use of stereotactic radiation to oligometastatic disease to further address if definitive radiation improves survival specifically among patients with breast cancer. BR002 only uses photon therapy, but the study advises a dose and fractionation scheme for bone metastases of 30 Gy in 3 fractions of 10 Gy, (EQD2 > 73, alpha/beta = 3) [10]. Most of the patients in our series received an EQD2 of 65 Gy(RBE) and have promising short-term local control, but pending the results of BR002, future patients may benefit from slightly higher equivalent doses (EQD2 > 70) to maximize the chance of long-term control.
